# Supplementary material for: Low-density lipoprotein receptor–related protein 1 (LRP1) as an auxiliary host factor for RNA viruses
Source: Life Sci Alliance. 2023 Apr 18;6(7):e202302005. doi: 10.26508/lsa.202302005 (PMC10114362; doi:10.26508/lsa.202302005)
Supplement: Supplementary file 1 [file LSA-2023-02005_TableS1.docx]

**Table S1.** Next Generation Sequencing (MiSeq, Solexa) iPCR primer sequences.
Index sequence is marked in red italics.

| Short name | Full name | Primer sequence | gDNA samples |
| --- | --- | --- | --- |
| DS | iPCR_DS | AATGATACGGCGACCACCGAGATCTACACGAGCCAGAACCAGAAGGAACTTGAC | All |
| A1 | iPCR_US_A_BC10 | CAAGCAGAAGACGGCATACGAGAT*GTCGTC*GTGACTGGAGTTCAGACGTGTGCTCTTC | Surviving retro-library / MseI |
| T1 | iPCR_US_T_BC10 | CAAGCAGAAGACGGCATACGAGAT*AGTCGTC*GTGACTGGAGTTCAGACGTGTGCTCTTCC | Surviving retro-library / NlaIII |
| A2 | iPCR_US_A_BC9 | CAAGCAGAAGACGGCATACGAGAT*ACTACT*GTGACTGGAGTTCAGACGTGTGCTCTTC | Surviving wild-type / MseI |
| T2 | iPCR_US_T_BC9 | CAAGCAGAAGACGGCATACGAGAT*AACTACT*GTGACTGGAGTTCAGACGTGTGCTCTTC | Surviving wild-type / NlaIII |
