# Supplementary material for: Low-density lipoprotein receptor–related protein 1 (LRP1) as an auxiliary host factor for RNA viruses
Source: Life Sci Alliance. 2023 Apr 18;6(7):e202302005. doi: 10.26508/lsa.202302005 (PMC10114362; doi:10.26508/lsa.202302005)
Supplement: Supplementary file 2 [file LSA-2023-02005_TableS2.docx]

**Table S2.** sgRNAs used for the CRISPR-hSpCas9 knock-out of the LRP1 gene in HuH-7 cells.

| **sgRNA** | **sequence** | **location** |
| --- | --- | --- |
| **LRP1_fwd** | **CACCGGCATGGACGGCTCAGATGAG** | **Exon 3** |
| **LRP1_rev** | **AAACCTCATCTGAGCCGTCCATGCC** |  |
