# Supplementary figures and images for: Low-density lipoprotein receptor–related protein 1 (LRP1) as an auxiliary host factor for RNA viruses
Source: Life Sci Alliance. 2023 Apr 18;6(7):e202302005. doi: 10.26508/lsa.202302005 (PMC10114362; doi:10.26508/lsa.202302005)

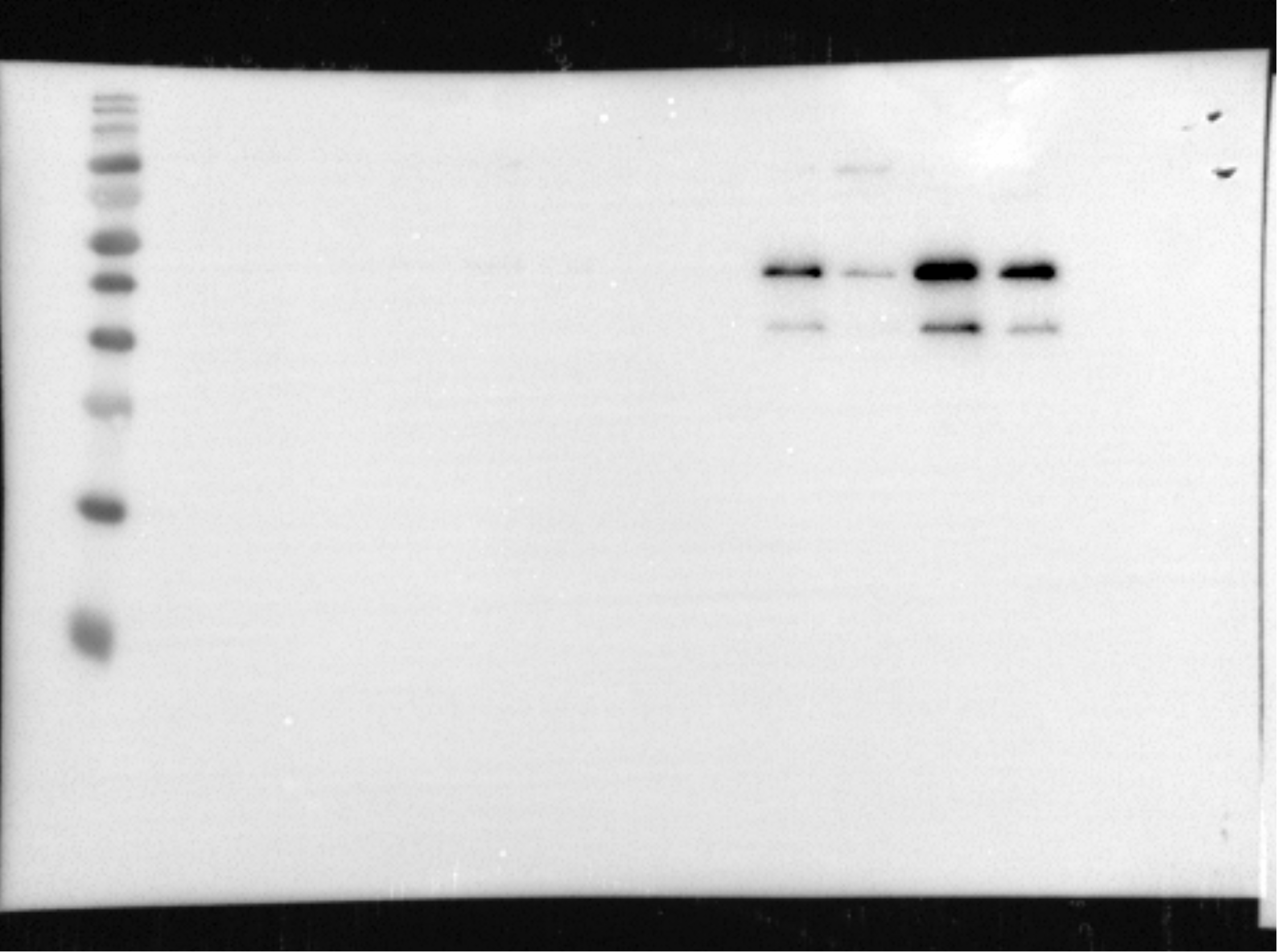

Supplement: Supplementary file 3 [file LSA-2023-02005_SdataF6.1.tif]

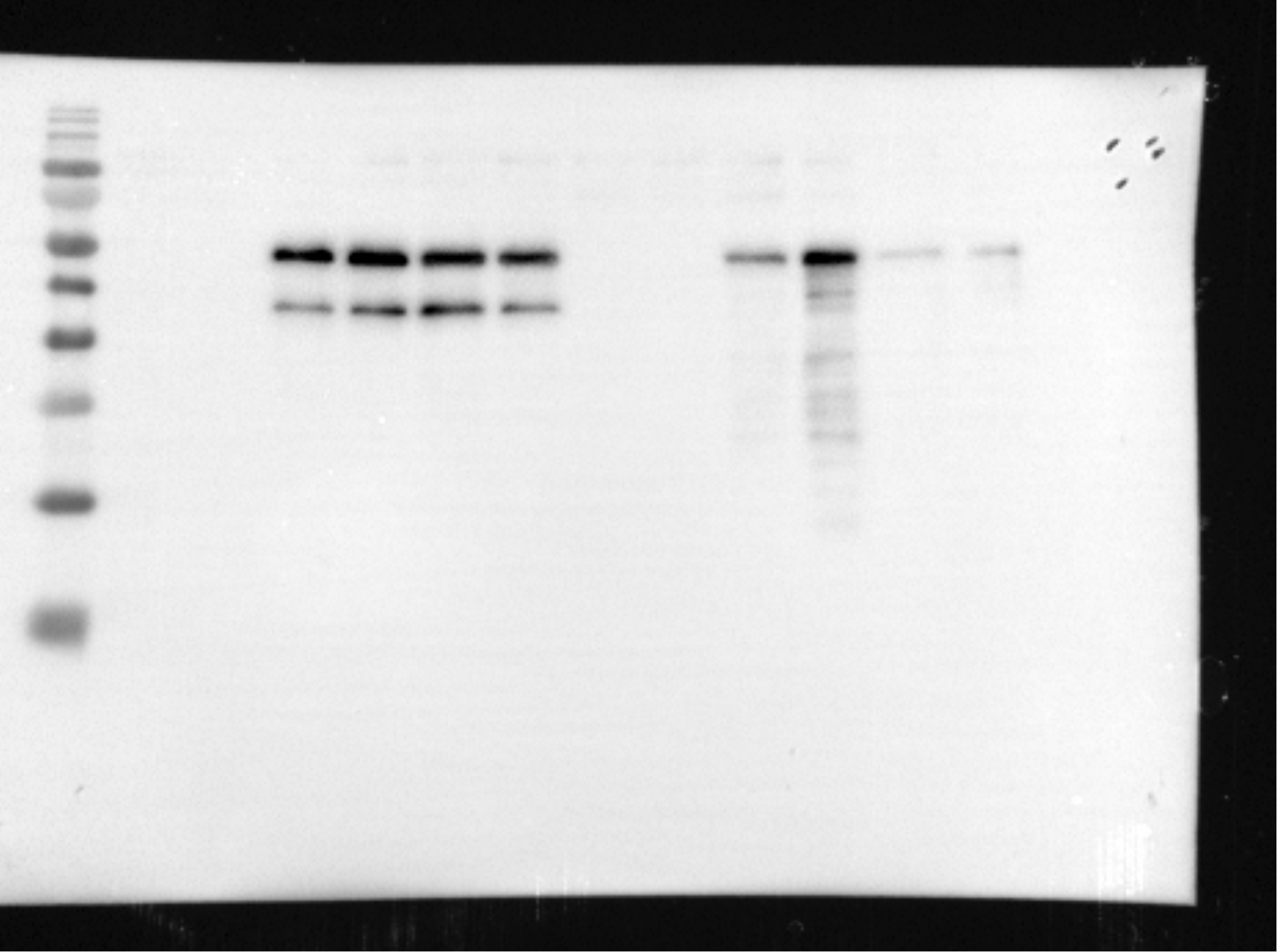

Supplement: Supplementary file 4 [file LSA-2023-02005_SdataF6.2.tif]

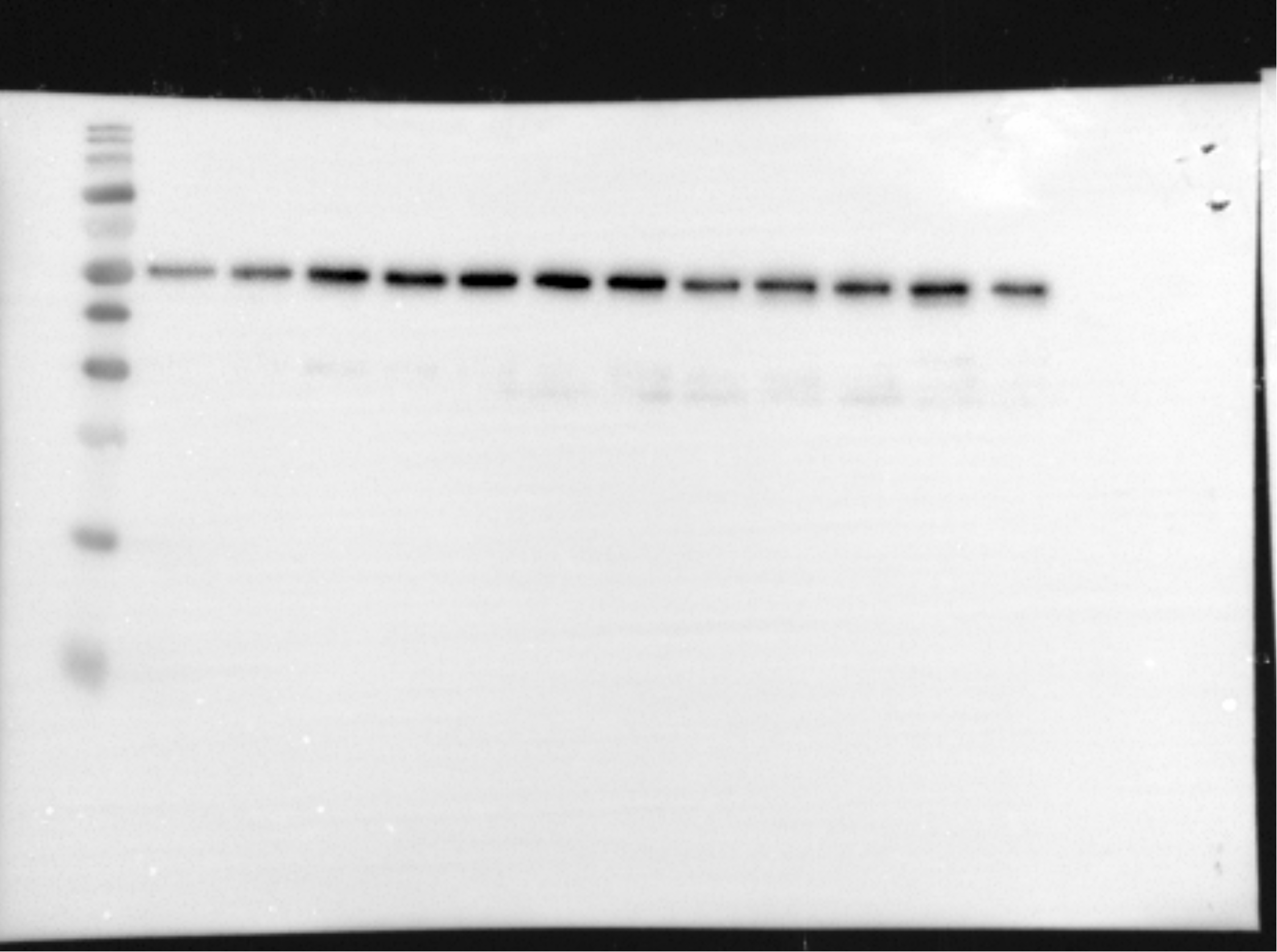

Supplement: Supplementary file 5 [file LSA-2023-02005_SdataF6.3.tif]

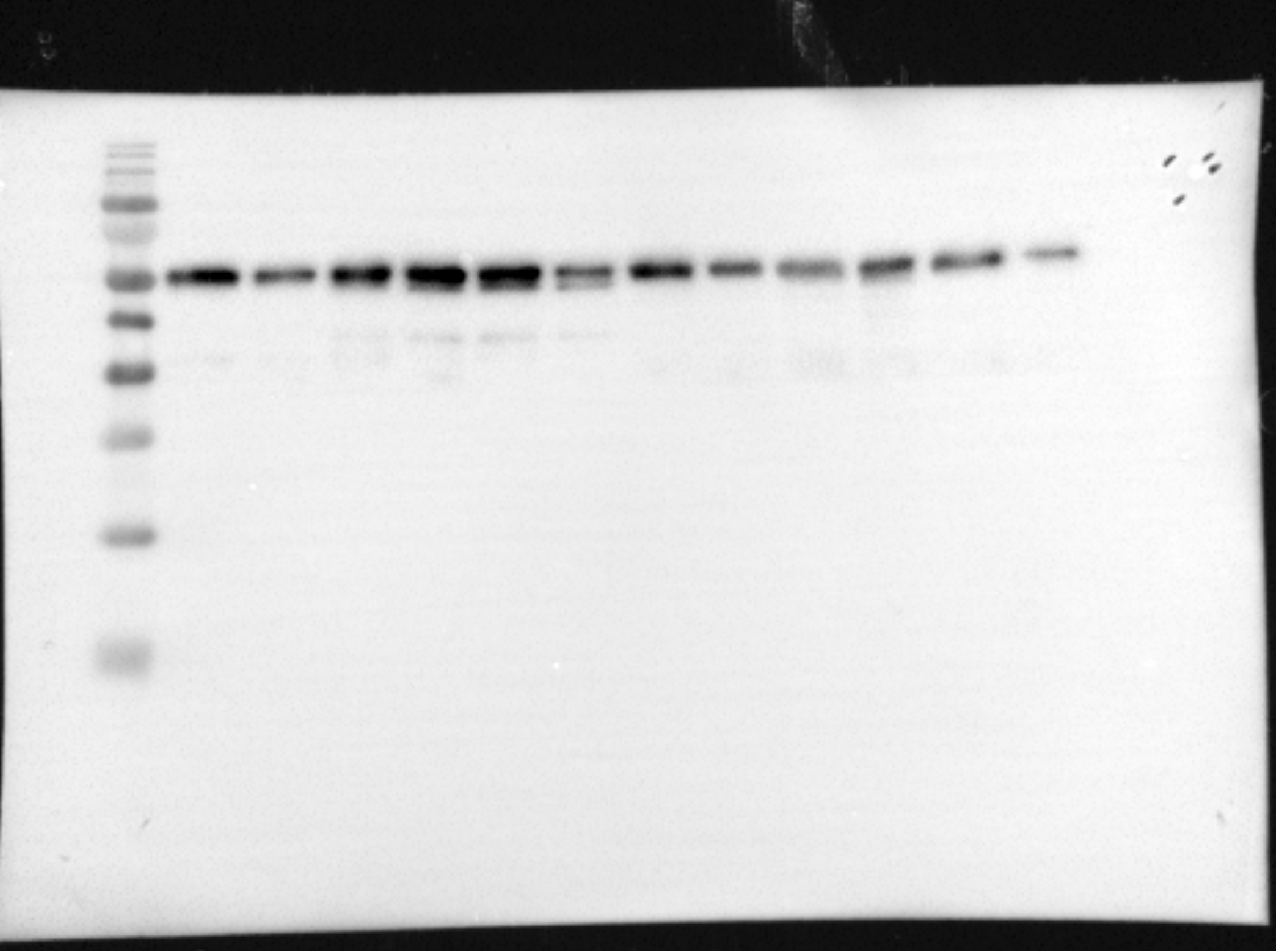

Supplement: Supplementary file 6 [file LSA-2023-02005_SdataF6.4.tif]
