# Supplementary material for: Low-density lipoprotein receptor–related protein 1 (LRP1) as an auxiliary host factor for RNA viruses
Source: Life Sci Alliance. 2023 Apr 18;6(7):e202302005. doi: 10.26508/lsa.202302005 (PMC10114362; doi:10.26508/lsa.202302005)
Supplement: Supplementary file 7 [file LSA-2023-02005_TableS3.docx]

**Table S3.** Primer and probe list for detection of virus RNA by qPCR.

| **Gene** | **Primer/Probe** | **Sequence** | **Ref.** |
| --- | --- | --- | --- |
| **EMCV gene 2B** | **EMCV 2B-F** | **ATGGGAAAATGTAAAAGAAACA** | **Qin *et al.*, 2018** |
|  | **EMCV 2B-R** | **GCATCACTGCTATTGTCA** |  |
|  | **EMCV 2B-P** | **6-FAM-AGCTGCACACATCTGCTCAA-BHQ1** |  |
| **LACV gene L** | **qPCR LACV fwd** | **AGGAAAACTCCTGAGAATATAACTA** | **This work** |
|  | **qPCR LACV rev** | **GGTATACAAACTGGTGGCGAT** |  |
|  | **qPCR LACV probe** | **6-FAM-CTTAAATTTGAAAATATGTCTAAAATCCAAACATACCCAGGC-BHQ-1** |  |
| **RVFV gene L** | **RVFL-2912fwdGG** | **TGAAAATTCCTGAGACACATGG** | **Bird *et al.*, 2007** |
|  | **RVFL-2981revAC** | **ACTTCCTTGCATCATCTGATG** |  |
|  | **RVFL-probe-2950** | **6-FAM-CAATGTAAGGGGCCTGTGTGGACTTGTG-BHQ1** |  |
| **SARS-CoV-2 gene E** | **E_Sarbeco_F** | **ACAGGTACGTTAATAGTTAATAGCGT** | **Corman *et al.*, 2020** |
|  | **E_Sarbeco_R** | **ATATTGCAGCAGTACGCACACA** |  |
|  | **E_Sarbeco_P1** | **6-FAM-ACACTAGCCATCCTTACTGCGCTTCG-BBQ** |  |
| **SFSV gene L** | **SFT FP** | **TCTGAGAACTGAGCTACAAGTG TTTATTA** | **Weidmann *et al.*, 2008** |
|  | **SFT RP** | **TTCCCATCTCTCTTCTGAAGAGTG** |  |
|  | **SFT P** | **6-FAM-AGGTCATAGACAGTATCATGAGAATTGCTAGGTG-BHQ-1** |  |
| **VSV gene N** | **VSV q-PCR fwd** | **GATAGTACCGGAGGATTGACGACTA** | **This work** |
|  | **VSV q-PCR rev** | **TCAAACCATCCGAGCCATTC** |  |
